# Human pluripotent stem cell-derived cardiomyocytes align under cyclic strain when guided by cardiac fibroblasts

**DOI:** 10.1063/5.0108914

**Published:** 2022-12-20

**Authors:** Dylan Mostert, Bart Groenen, Leda Klouda, Robert Passier, Marie-Jose Goumans, Nicholas A. Kurniawan, Carlijn V. C. Bouten

**Affiliations:** 1 Department of Biomedical Engineering, Eindhoven University of Technology, PO Box 513, 5600 MB Eindhoven, The Netherlands; 2 Institute for Complex Molecular Systems (ICMS), PO Box 513, 5600 MB Eindhoven, The Netherlands; 3 Department of Applied Stem Cell Technologies, University of Twente, Enschede, The Netherlands; 4 Department of Anatomy and Embryology, Leiden University Medical Centre, Leiden, The Netherlands; 5 Department of Cell and Chemical Biology and Center for Biomedical Genetics, Leiden University Medical Centre, Leiden, The Netherlands

## Abstract

The myocardium is a mechanically active tissue typified by anisotropy of the resident cells [cardiomyocytes (CMs) and cardiac fibroblasts (cFBs)] and the extracellular matrix (ECM). Upon ischemic injury, the anisotropic tissue is replaced by disorganized scar tissue, resulting in loss of coordinated contraction. Efforts to re-establish tissue anisotropy in the injured myocardium are hampered by a lack of understanding of how CM and/or cFB structural organization is affected by the two major physical cues inherent in the myocardium: ECM organization and cyclic mechanical strain. Herein, we investigate the singular and combined effect of ECM (dis)organization and cyclic strain in a two-dimensional human *in vitro* co-culture model of the myocardial microenvironment. We show that (an)isotropic ECM protein patterning can guide the orientation of CMs and cFBs, both in mono- and co-culture. Subsequent application of uniaxial cyclic strain—mimicking the local anisotropic deformation of beating myocardium—causes no effect when applied parallel to the anisotropic ECM. However, when cultured on isotropic substrates, cFBs, but not CMs, orient away from the direction of cyclic uniaxial strain (strain avoidance). In contrast, CMs show strain avoidance via active remodeling of their sarcomeres only when co-cultured with at least 30% cFBs. Paracrine signaling or N-cadherin-mediated communication between CMs and cFBs was no contributing factor. Our findings suggest that the mechanoresponsive cFBs provide structural guidance for CM orientation and elongation. Our study, therefore, highlights a synergistic mechanobiological interplay between CMs and cFBs in shaping tissue organization, which is of relevance for regenerating functionally organized myocardium.

## INTRODUCTION

The function of the myocardium is highly dependent on its unique structural organization of cells and extracellular matrix (ECM). In the healthy human myocardium, cardiomyocytes (CMs) and quiescent cardiac fibroblasts (cFBs) are arranged as dense, aligned cellular aggregates surrounded by an anisotropic collagen matrix. This spatial arrangement enables electrical and mechanical coupling between CMs and facilitates their synchronous, coordinated contraction.^1–3^ Following ischemic cardiac injury, such as myocardial infarction (MI), billions of CMs die. The ischemia and subsequent inflammatory response at the site of injury initiate the activation and differentiation of cFBs toward myofibroblasts. These cells not only aid in replacing the damaged tissue with abundant ECM but also mediate adverse remodeling of the anisotropic myocardium into a disorganized fibrotic scar tissue. In addition, the myofibroblasts secrete profibrotic and hypertrophic cytokines, advancing the progression of adverse remodeling in the continuously beating myocardium and ultimately leading to heart failure.^4–10^

The relevance of anisotropic cellular organization for adequate myocardial function has been demonstrated using well-defined *in vitro* systems. For instance, aligned CMs on two-dimensional (2D) substrates demonstrated improved calcium handling and contractile properties compared to randomly oriented CM monolayers.^11^ In three-dimensional (3D) cardiac microtissues, aligned CMs and ECM were found to generate a homogeneous and coordinated contraction, which was not observed in isotropic tissues.^12^ Furthermore, alignment of mesenchymal stem cells, induced by gelatin microgrooves, supported differentiation toward the myocardial lineage.^13^ These studies suggest that restoring tissue anisotropy may represent a critical step in the functional regeneration of damaged myocardium. Yet, until today, cardiac regenerative strategies have largely focused on replenishing CMs,^14–16^ while the restoration of the myocardial structural organization is largely overlooked.

*In vivo*, the mechanically active myocardium presents cyclic deformations—due to cardiac beating—as well as structural cues—imposed by collagen fibers—to the CMs and cFBs. Such microenvironmental biophysical cues are known to affect cell alignment by mediating the anisotropic organization of their internal structures.^17^ *In vitro*, anisotropic structural guidance cues can be mimicked using parallel micro-grooves, adhesive protein patterns, or aligned scaffold fibers, demonstrating that both cFBs^18–20^ and CMs^11,19–22^ can sense this 2D micro-environment and align with the anisotropy presented—a phenomenon termed contact guidance. When subjected to uniaxial cyclic strain, adherent cells may (re)orient toward the direction (almost) perpendicular to the strain—a phenomenon called strain avoidance. Dynamic reorganization and remodeling of actin stress fibers in combination with a nucleo-cytoskeletal connection are believed to play an important role in the cell type-dependent response to uniaxial cyclic strain (mechanoresponse).^23–25^ While cFBs are consistent in their strain avoidance behavior,^26,27^ CMs show inconsistent responses,^27–31^ probably related to differences in their contractile state and actin organization.^32,33^ To date, CM mechanoresponse remains largely unexplored, contributing to the controversy about how CMs respond to cyclic strain. Moreover, little is known about the collective organization of physiological and pathological combinations of CMs and cFBs, and how this is guided or disrupted by structural and mechanical cues in the myocardium.^22^ A better understanding of how biophysical cues in the myocardium can contribute to the singular and collective organization of CMs and cFBs represents a key step for improving *in vivo* myocardial regeneration, including cell-based therapies.

In this study, we address this knowledge gap by emulating the fibrous ECM organization and beating of the myocardium in a 2D *in vitro* model of the human myocardial microenvironment. Human fetal epicardial cell derived cFBs and human pluripotent stem cell derived cardiomyocytes (CMs) were cultured on (an)isotropic ECM protein patterns and subsequently subjected to uniaxial cyclic strain. By systematically examining the cellular response of monocultures as well as co-cultures of (varying ratios of) CMs and cFBs to singular and combined environmental cues, this approach provided novel insights into the interplay between the two cell types in regulating organization in physiological and pathological myocardial tissue compositions.

## RESULTS

### ECM patterning guides CM and cFB orientation

**FIG. 1.**
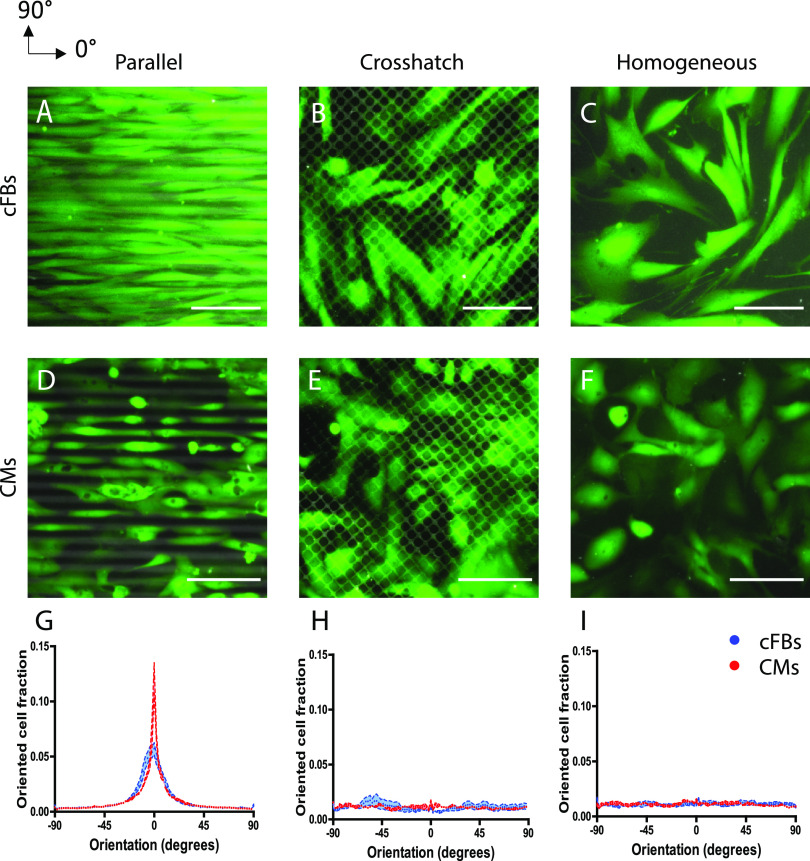
Fibronectin (FN) patterns obtained by micro-contact printing onto deformable PDMS substrates guide cell body organization of cFBs and CMs. (a)–(f) Representative fluorescent images showing FN patterns (TRITC-fibronectin, gray) and cFBs (a)–(c) and CMs (d)–(f) (calcein AM, green). (g)–(i) Frequency distribution graphs display the cell orientation with respect to the horizontal direction (0°) for cFBs (blue) and CMs (red). Scale bar indicates 100 *μ*m.

Structurally, the myocardium is a fibrous tissue, implying that cardiac cells receive structural cues from contact with ECM fibers. Similar anisotropic topographical or structural cues from the substrate, such as micropatterns, microfabricated grooves, or collagen fibers, have been shown to guide the orientation of CMs and cFBs—a phenomenon termed “contact-guidance.”^11,34,35^ Here, to mimic the local anisotropic structure of the healthy myocardium and to allow application of mechanical strain, we created fibronectin (FN) adhesive patterns on deformable polydimethylsiloxane (PDMS) membranes using previously established microcontact printing methods (Fig. 1).^36^ Two types of patterns were produced: (i) parallel lines with a linewidth (10 *μ*m), identical to the inter-line spacing [Figs. 1(a) and 1(d)], corresponding to the physiological fiber size of perimysial collagen in the myocardium,^37^ and (ii) crosshatch patterns [orthogonal linear features of 5 *μ*m linewidth and 10 μm spacing, Figs. 1(b) and 1(e)] to mimic isotropic ECM. The linewidth for the crosshatch pattern was set to be half that of the parallel line patterns to achieve comparable area density for cell adhesion on the two pattern types. Homogeneously FN-printed PDMS using an unpatterned microstamp served as control substrate [Figs. 1(c) and 1(f)]. All FN patterns remained visually intact and well defined throughout the experimental procedures of 48 h of strained or static cell culture (Fig. S1).

Orientation of cells was evaluated using cytoplasmic calcein AM staining, which circumvents drawbacks with cytoskeleton-based staining due to the substantially different cytoskeletal organizations in CMs and cFBs and serves as optimal start for the quantification of cell orientation and shape in the same wells over time. Within 24 h of seeding on the printed ECM patterns, both cFBs [Fig. 1(a)] and CMs [Fig. 1(d)] showed a clear contact guidance behavior. cFBs were aligned in the direction of the parallel lines and consistently displayed an elongated morphology on all FN patterns with an aspect ratio (AR) of 4.5 ± 2.9 (n = 30) on the parallel patterns and 3.5 ± 2.7 (n = 30) on crosshatch patterns. Alignment along the parallel lines was also found for the CMs, although the CMs showed morphological heterogeneity and formed small aggregates on the printed ECM patterns. Such aggregations are often found in contracting CMs cultures and involve cell–cell interactions,^38^ possibly influencing the amount of CM adhesion to the parallel protein patterns. However, CM density on the substrates did not change during the experiments, suggesting that the vast majority of CMs maintained cell–ECM interactions with the FN patterns. The CMs exhibited an AR of 5.3 ± 1.8 (n = 30) on parallel lines and 2.1 ± 1.0 (n = 30) on crosshatch patterns. The AR found on the crosshatch protein patterns is in line with previous studies using hPSC-CMs^39^ and is lower than that for adult CMs *in vivo*,^38^ which has been attributed to the lower maturity state of hPSC-CMs.

Previously, it has been shown that anisotropic ECM induced the alignment of cFBs,^18–20^ hPSC-CMs,^40–43^ and neonatal rat CMs.^13,42,44^ Consistent with these reports, quantification of cell orientation showed a peak in the direction of the parallel FN lines (0°) and random distribution on crosshatch and homogeneous FN for both cFBs and CMs [Fig. 1(g)–1(i)]. Notably, a direct comparison between the two cell types on the same, well-defined ECM patterns, revealed that CMs exhibited more pronounced anisotropic orientation than the cFBs [Fig. 1(g)]. This is possibly explained by the smaller cell size of the CMs (110 ± 26 *μ*m longitudinal axis) compared to cFBs (242 ± 59 *μ*m longitudinal axis). Indeed, Buskermolen *et al.* showed that cell size is an important determinant of cell alignment on microscale ECM patterns and found that the alignment of cardiomyocyte progenitor cells (∼100 *μ*m length) occurred at smaller ECM pattern size compared to that of myofibroblasts (∼200 *μ*m length).^45^ Taken together, these experiments confirmed that ECM patterning is an effective approach to exploit contact guidance to direct the alignment response of both CMs and cFBs.

### cFB cultures, but not CM cultures, show strain avoidance in response to uniaxial cyclic strain

**FIG. 2.**
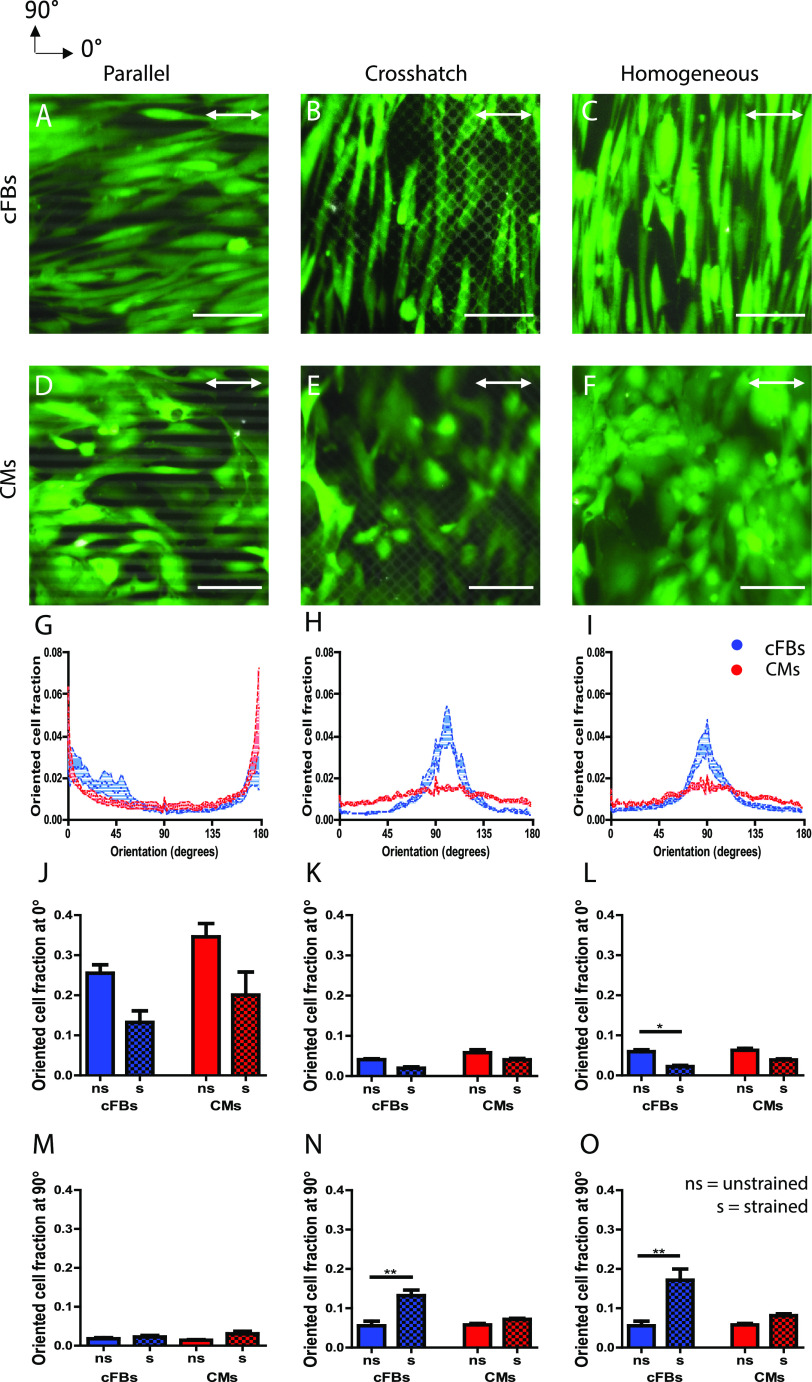
CM and cFB orientations are dominated by contact guidance on anisotropic patterns, while for cFBs, strain-avoidance is found on disorganized and homogeneous FN patterns. (a)–(f) Representative fluorescent images showing FN patterns (TRITC-fibronectin, gray) and cFBs (a)–(c) and CMs (d)–(f) (calcein AM, green) after 48 h of dynamic culture. The direction of the uniaxial cyclic strain is indicated by the white arrow. (g)–(i) Frequency distribution graphs comparing the response of cFBs (blue) and CMs (red) display a clear peak at 90° for cFBs on crosshatch and homogeneous ECM while this is not observed for CMs. (j)–(o) Oriented cell fractions at 0° ± 5° and 90° ± 5°, showing significant reorganization of the cFBs (blue) upon administration of uniaxial cyclic strain, whereas this is not observed for CMs (red). ns: non-strained; s: strained. Scale bar indicates 100 *μ*m. ^*^P <0.05; ^**^P <0.01.

Mechanically, the myocardium resident cells experience continuous cyclic mechanical strain due to cardiac beating. Thus, we sought to investigate the orientation response of CMs and cFBs to cyclic strain in the presence of guiding ECM structures mimicked by protein patterns. For several cardiovascular cell types, studies have shown a synergistic effect of structural guidance cues and uniaxial cyclic strain when the cyclic strain direction is presented perpendicular to the anisotropic ECM structures.^46,47^ In contrast, we now asked whether ECM alignment and cyclic strain present competing cues when they are applied in the same direction, similar to the mechanical microenvironment in the myocardium. To this end, we seeded CMs and cFBs on printed ECM patterns that were subsequently subjected to uniaxial cyclic strain at a physiological strain magnitude of 10% and a frequency of 0.5 Hz (Fig. 2). In response to such strains, several adherent cell types have been reported to orient perpendicular to the direction of applied cyclic strains—a phenomenon called “strain avoidance.”^24,25,48,49^ On the anisotropic ECM patterns, however, both the CMs and cFBs showed only a slight disruption of cellular alignment without a complete strain avoidance response within the 48 h of cyclic strain [Figs. 2(a), 2(d), and 2(g)]. Quantification of the cell orientation demonstrated a slight decrease in the fraction of cells oriented at 0° ± 5° (contact-guided response) and a lack of alignment in the direction perpendicular to the strain at 90° ± 5° (strain avoidance response) [Figs. 2(j) and 2(m)], supporting this dominance of cellular contact guidance over strain avoidance. However, when the starting point was a dis- or nonorganized cellular organization (on crosshatch ECM patterns and homogeneous FN), the cFBs displayed a clear strain avoidance response, as most cells oriented toward 90° [Figs. 2(b)–2(c), 2(h)–2(i), blue]. This is consistent with the reported response of cFBs on homogeneous ECM.^26,50^ In contrast, uniaxial cyclic strain failed to induce orientation of the CMs away from the direction of the applied dynamic strain, even on dis- or nonorganized ECM [Figs. 2(e) and 2(f), 2(h)–2(i), red]. This dispersity between cell types is further highlighted by the significant increase in the oriented cell fractions at 90° ± 5° for cFBs and the absence of (re)orientation for the CMs [Figs. 2(n) and 2(o)]. These findings suggest that cFBs and CMs have different intrinsic abilities to sense and respond to cyclic strain.

### Cardiac co-cultures of varying cell ratios organize in response to uniaxial cyclic strain

Given the stark contrast between the collective cellular responses of CMs and cFBs to environmental cues, we next asked how their difference in mechanoresponsiveness would dictate the organization of the intracellular F-actin and sarcomeres (α-actinin) structures in myocardial co-cultures. To assess how co-culture composition influences the overall mechanoresponse, we created co-cultures of CMs and cFBs with either 70:30 (CM-rich) or 30:70 (cFB-rich) cell seeding ratio. *In vivo*, adult CMs account for ∼70% of myocardial volume, although they comprise ∼30% of myocardial cell number.^51,52^ However, the cellular volume of adult CMs largely differs from the CM volume used in this study, suggesting that co-cultures consisting of ≥30% cFBs are of relevance to mimic the cellular distribution of the myocardium. We note that impurities in the CM culture and an increase in number of viable cFBs led to an increased number of non-myocyte cells in the co-culture during the experimental procedure, resulting in co-cultures with 55.3% ± 16.5% α-actinin positive cells (indicative for CMs) for the CM-rich co-cultures and 18.7% ± 9.4% for the cFB-rich co-cultures after 48 h [Fig. S2(a)]. In other words, although the cell composition changed throughout the experimental procedure, the number of CMs was always significantly higher in the CM-rich co-culture. Cell viability remained constant between experimental conditions and time points [Figs. S2(b)–S2(e)].

**FIG. 3.**
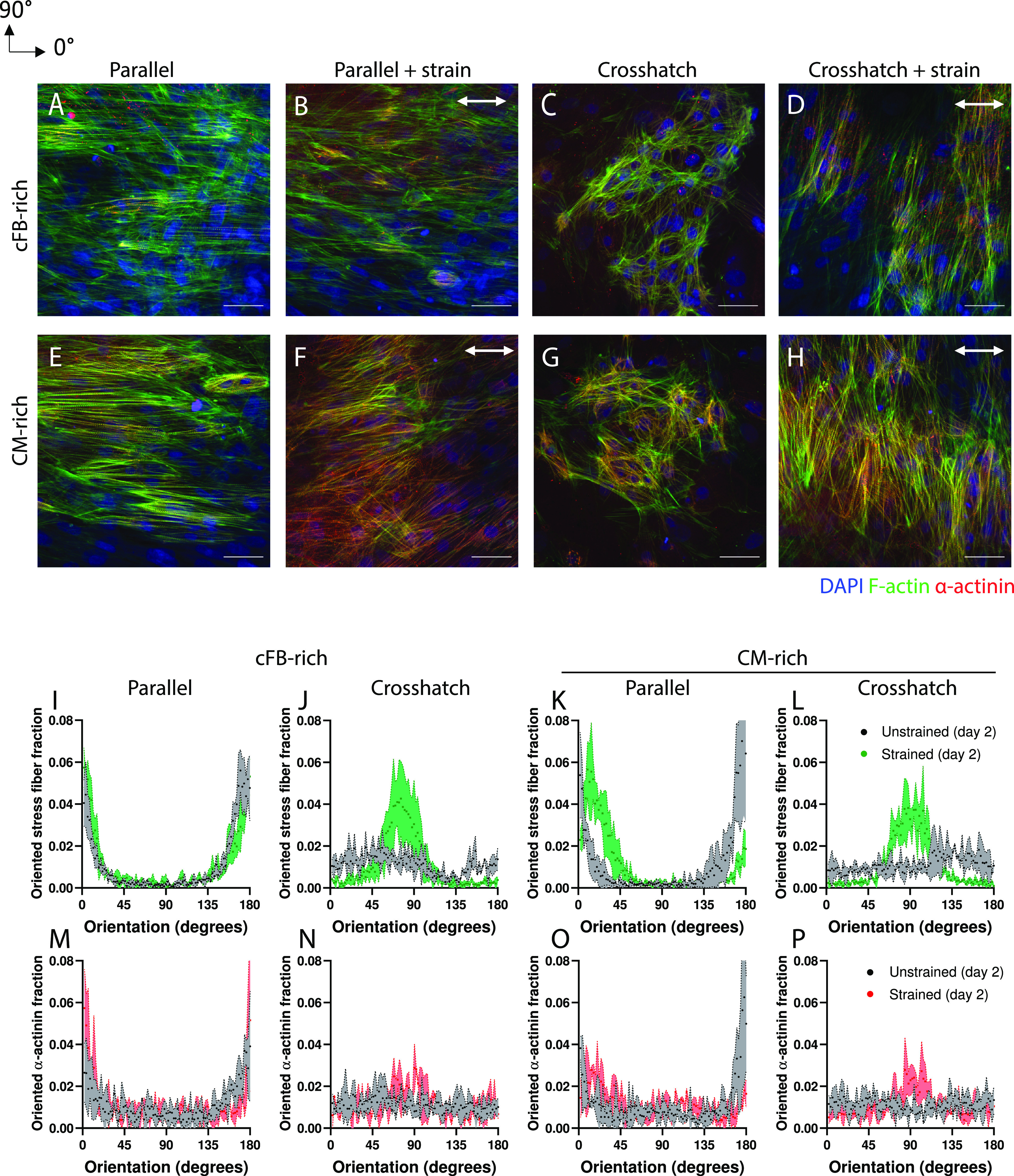
cFB-rich and CM-rich co-cultures show modest strain avoidance behavior in response to uniaxial cyclic strain (a)–(h) representative fluorescent images of 4',6-Diamidino-2-phenylindole dihydrochloride (DAPI, blue), F-actin (green), and α-actinin (red) in the co-cultures after 48 h of static or dynamic culture on printed FN patterns. The direction of the uniaxial cyclic strain is depicted with the white arrow. Scale bar indicates 50 *μ*m. (i)–(p) Frequency distributions histograms comparing the orientation response of F-actin (green) and α-actinin (red) under strained and unstrained conditions. Scale bar indicates 50 *μ*m.

Under static culture conditions, both co-cultures demonstrated cellular alignment on the linear ECM patterns [Figs. 3(a) and 3(e)] and a random cellular orientation on the crosshatch ECM patterns [Figs. 3(c) and 3(g)], similar to the response of monocultures of cFBs and CMs. This is further demonstrated by the frequency distribution histograms for F-actin and sarcomere orientation, both showing a peak ∼0° for the parallel patterns and no preferred orientation for the crosshatch patterns [Figs. 3(i)–3(p)].

To address how the mechanical environment in the myocardial niche, where ECM guidance and cyclic strain together influence the organization of cardiac cells, affects the organization of cardiac co-cultures, we studied the combinatory effect of uniaxial cyclic strain and (an)isotropic ECM on cFB-rich and CM-rich co-cultures. When uniaxial cyclic strain was applied in the same direction as the anisotropic ECM, both co-cultures lacked a distinct strain avoidance behavior [Figs. 3(b), 3(f), 3(i), 3(k), 3(m), and 3(o)], although a slight disruption in preferred orientation was found for the CM-rich co-culture in both F-actin and sarcomere organization [Figs. 3(k) and 3(o)]. These results show that contact-guided cellular organization of cardiac co-cultures cannot be easily disrupted by cyclic strain induced strain avoidance, at least with the pattern dimensions and strain protocols used in this study.

Next, we analyzed whether cyclic strain could overpower the disorder that results from disorganized ECM. To do so, the cardiac co-cultures were strained after culture on crosshatch patterns. Both the CM-rich and cFB-rich co-cultures showed anisotropic organization of the cellular monolayers after 48 h of dynamic culture [Figs. 3(d) and 3(h)], together with realignment of both their F-actin and sarcomere structures [Figs. 3(j), 3(l), 3(n), and 3(p)]. This was unexpected, given that CM monocultures did not show such strain avoidance response. Together, these findings suggest that cFBs guide the orientation of CMs when they align in response to uniaxial cyclic strain, either by direct cell–cell contact or via paracrine signaling. Moreover, these results suggest that uniaxial cyclic strain can be used to induce linear organization of cardiac cells on crosshatch ECM patterns.

To assess the specific contribution of the CMs to the collective reorientation response of the co-cultures, we used a double fluorescent hPSC reporter of mRubyIIACTN2 and GFP-NKX2.5 (DRRAGN).^53^ This cell line demonstrates identical behavior as our earlier findings above, both as monoculture and in co-culture with cFBs (Fig. S3). Specifically, quantification of stress fiber (cFBs) and sarcomere (CM) orientation demonstrated the lack of a strain avoidance response in CMs, which is typical for the cFBs [Figs. S3(d) and S3(i)]. Assessment of nuclear orientation and aspect ratio showed a significant increase in nuclear aspect ratio for cFBs when strain was applied as opposed to unstrained cFBs, which was not the case for CMs. Moreover, clear nuclear alignment was found in the direction perpendicular to the strain for cFBs, which was not observed for CMs when uniaxial cyclic strain was applied [Figs. S3(e) and S3(j)].

**FIG. 4.**
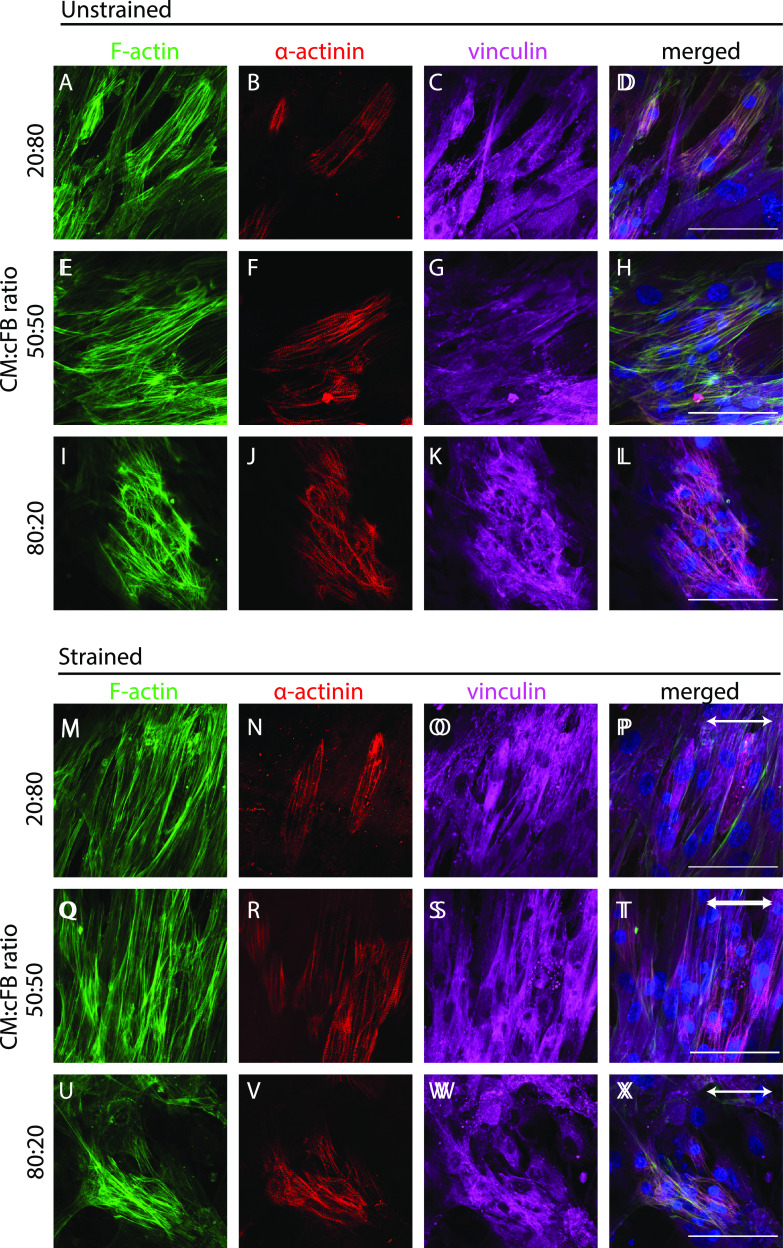
Uniaxial cyclic strain induces sarcomere alignment in CMs when in co-culture with a threshold value of cFBs. (a)–(l) Representative fluorescent images of F-actin (green), α-actinin (red), and vinculin (magenta) in co-cultures with varying seeding ratios after 48 h of static culture on homogeneous FN. (m)–(x) Representative fluorescent images of the co-cultures after 48 h of dynamic culture on homogeneous FN. The direction of the uniaxial cyclic strain is depicted with the white arrow. Scale bar indicates 100 *μ*m.

**FIG. 5.**
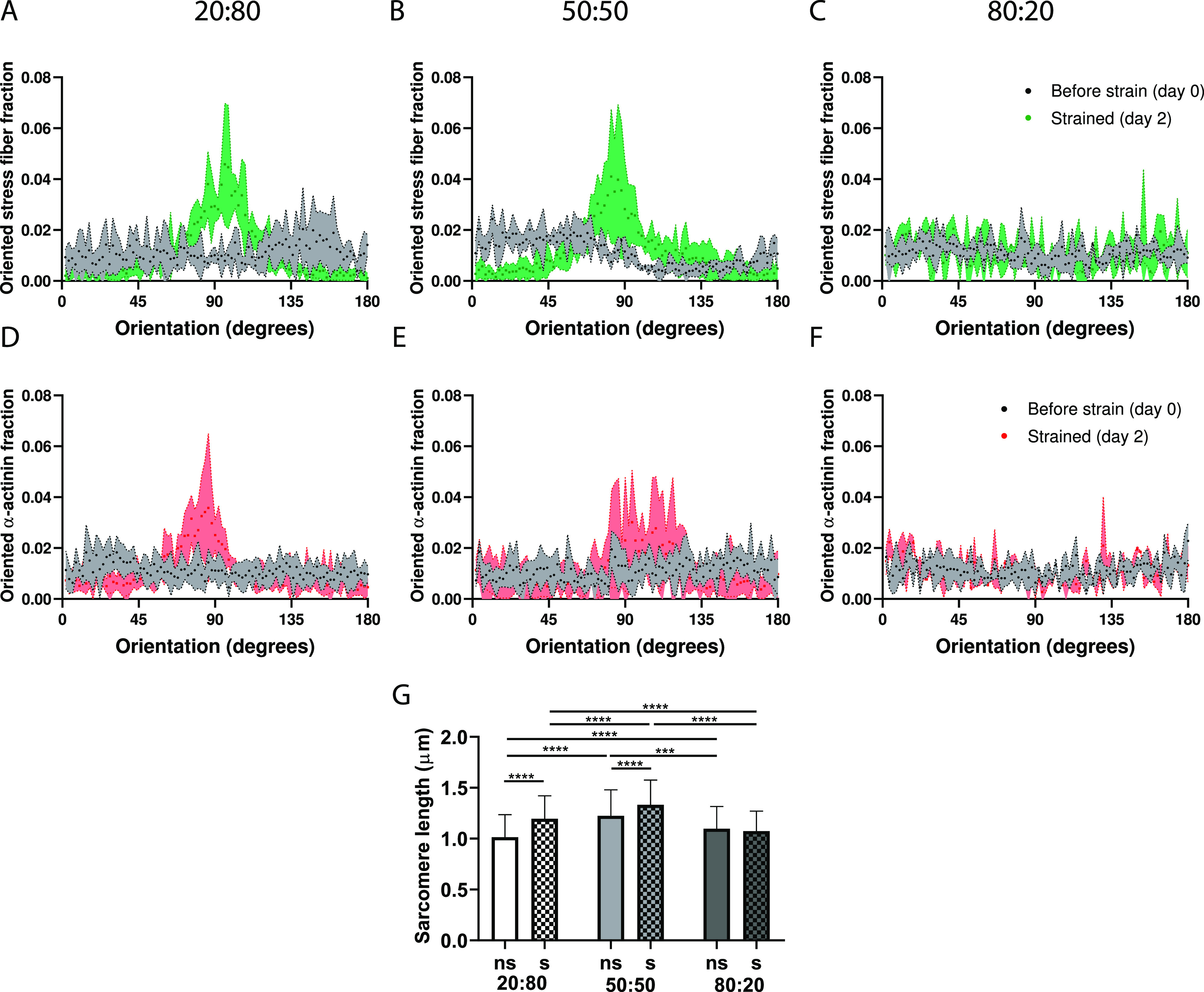
Uniaxial cyclic strain induces sarcomere alignment (CMs) and F-actin alignment (cFBs) when in co-culture with a threshold value of cFBs. (a)–(f) Frequency distributions histograms comparing the orientation response of F-actin (green) (a)–(c) and α-actinin (red) (d)–(f) before and after 48 h of uniaxial cyclic strain. (g) Sarcomere length was significantly increased upon cyclic strain administration in the 20:80 and 50:50 co-cultures (n = 500 sarcomeres per condition). ns: non-strained, s: strained. ^*^P <0.05, ^**^P <0.01, ^***^P <0.001, ^****^P <0.0001.

To assess the amount of cFBs necessary to induce the strain-induced alignment of CMs, we used this reporter cell line to systematically create co-cultures with CM-cFB ratios of 20:80, 50:50, 80:20 and subject them to uniaxial cyclic strain for 48 h on homogenous FN. Qualitative assessment of α-actinin expression by immunofluorescent staining demonstrated aligned sarcomere structures in the direction almost perpendicular to the strain in the 20:80 and 50:50 co-cultures [Figs. 4(n) and 4(r)], similar to what was observed in the 70:30 and 30:70 co-cultures. Surprisingly, both CM and cFBs did not reorient in the 80:20 co-culture after strain application [Figs. 4(u)–4(x)]. Quantification of F-actin and sarcomere orientation, which can be linked to the direction of CM contraction, indeed, showed a distinct preferred sarcomere orientation in the 90° direction in contrast to unstrained controls [Figs. 5(a), 5(b), 5(d), and 5(e)]. In addition, as opposed to the more rounded morphologies in CM monocultures and the 80:20 co-culture [Figs. 4(i)–4(l) and 4(u)–4(x)], CMs demonstrated elongated morphologies and sarcomere alignment both under dynamic and static conditions when enough cFBs were present. Notably, a lack of strain-induced alignment was found for CMs in the 80:20 culture [Figs. 5(c) and 5(e)], suggesting a threshold of cFBs, that is, needed in the co-culture to induce strain-induced alignment of CMs.

Since sarcomere length is greater with increased CM contractility^54–56^ and can, therefore, serve as a preliminary assessment of CM function, we quantified sarcomere length in the co-cultures of various ratios under static and strained conditions [Fig. 5(g)]. Although the exact values of sarcomere length obtained using this approach might not be as accurate as quantified with other techniques,^57,58^ our results clearly indicate that cardiomyocytes in the 20:80 and 50:50 co-cultures demonstrated sarcomeres of greater length when cyclic strain was applied as opposed to unstrained conditions, indicative of an increased contraction force. Increased sarcomere length with cyclic strain is in line with Zhang *et al.* who reported lengthening of sarcomeres when engineered heart tissues were loaded cyclically.^59^ Interestingly, sarcomere length was found highest in the 50:50 co-culture, both in the strained and unstrained condition, suggesting that a 1:1 ratio is more favorable for the CM contractile properties as opposed to 1:5 and 5:1. Future studies should be done to probe this further at the cardiac functional level.

Together, these results suggest a threshold of the cFB number (>30%) required for inducing strain avoidance in CMs as well as aligned sarcomere structures with greater sarcomere length, indicative of CM maturity.^60,61^

### Strain avoidance of CMs in the presence of cFBs is not mediated by paracrine signaling or N-cadherin

We next asked what mechanisms are underlying the strain avoidance of CMs when in co-culture with a threshold value of cFBs. In particular, we wondered if the observed effect could be explained by paracrine signaling of the cFBs or by direct cell–cell communication via adherens junctional protein N-cadherin. To assess whether the impact of cFBs on CMs alignment could be attributed to paracrine factors, conditioned medium was collected from cFB monocultures after 48 h of uniaxial cyclic strain and added to the CMs before strain application. After 48 h cyclic strain of CMs in the conditioned medium, no significant differences in cell orientation were found (p = 0.28) in comparison to untreated monocultures of CMs [Figs. S4(a), S4(b), S4(e), and S4(f)]. Moreover, limited sarcomere formation was observed in the CMs in the conditioned medium, similar to the CM monocultures, suggesting that the improved maturation state of CMs in co-culture with cFBs is not attributable to paracrine signaling [Figs. S4(c) and S4(d)].

To investigate whether adherens-junction-mediated cell–cell communications play a role in the strain-induced alignment of CMs in cardiac co-cultures, we analyzed the expression and localization of N-cadherin (Fig. S5). While N-cadherin expression was clearly observed at the cell membrane between CMs, N-cadherin was absent between cFBs, and merely cytoplasmic N-cadherin was observed in CMs when in direct contact with cFBs, lacking the clear membrane bound N-cadherin expression found between CMs [Figs. S5(g), S5(j), and S5(m)]. Moreover, no preferential organization of N-cadherin mediated cell–cell interactions was found when CMs are organized via cyclic strain [Figs. S5(n) and S5(m)]. These results suggest that the strain-induced alignment of CMs in co-culture with cFBs is caused neither by paracrine signaling nor by N-cadherin mediated cell–cell communication between the CMs and cFBs. This is in line with a recent report showing a lack of Cx43 and N-cadherin-mediated cell–cell communication in the co-culture organization of neonatal rat CMs and cFBs.^27^ Future experiments using targeted inhibition of Cx43 and N-cadherin in co-cultures under cyclic strain together with transcriptional analysis should be performed to validate these findings.

## DISCUSSION

**FIG. 6.**
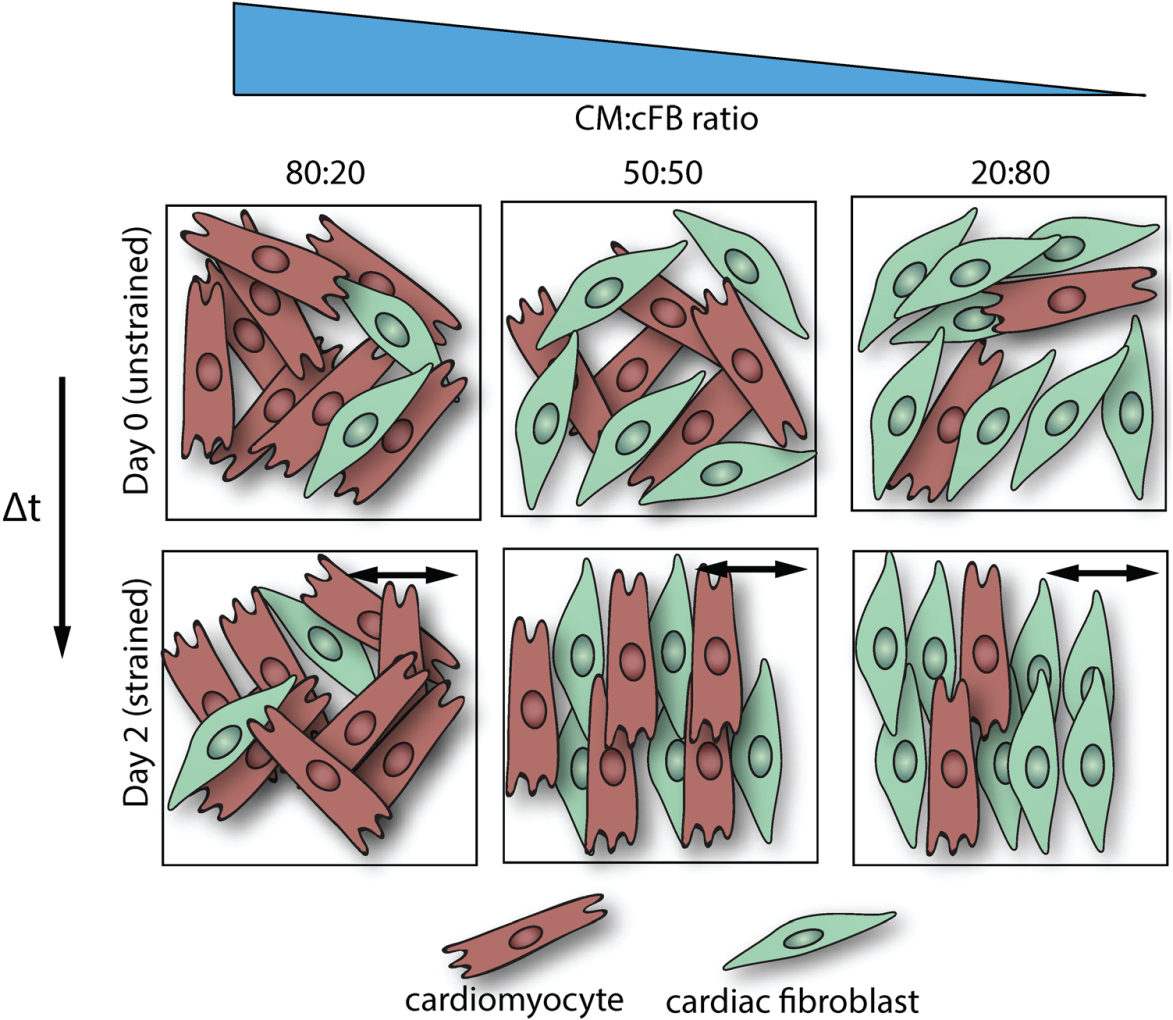
Conceptual schematic representation of the changes in cFB orientation upon the application of 48 h of uniaxial cyclic strain and the proposed link to CM alignment. Top row: co-cultures of various seeding densities show random organization under static culture conditions. Bottom row: upon cyclic strain application, strain-avoidance behavior is found for cFBs, which, in turn, serve as guidance cues for CM orientation. We propose the formation of anisotropic guidance cues, resulting from the strain-avoidance response of cFBs, which steer CM alignment and sarcomere organization in the direction almost perpendicular to the applied cyclic strain. The black arrow depicts the direction of the cyclic strain.

In our quest to develop strategies to regenerate cardiac anisotropy, we aimed to gain fundamental understanding of how biophysical cues from the myocardium contribute to shaping and disrupting anisotropy by organizing the main cell types in the cardiac wall. Specifically, we studied the collective reorganization response of cFBs and CMs, derived from induced pluripotent stem cells, under the influence of cyclic strain and ECM protein patterns to model both the cellular composition of myocardial tissue in health and disease and the dynamic CM microenvironment. Notably, most studies that address the effect of biophysical cues on cardiac cell organization have focused solely on monocultures,^11,18,29,31,62^ while crosstalk between cFBs and CMs is found to be critical for myocardial function.^63,64^ To this end, we used a 2D *in vitro* approach of micropatterned ECM protein patterns on deformable substrates, which allowed controlled presentation of structural cues and mechanical strains to dissect how these independently and simultaneously affect the organization of monocultures and co-cultures. We show that CMs in co-culture with ≥30% cFBs exhibit a collective strain avoidance response concurrent with sarcomere lengthening and sarcomere alignment in the same direction, which was not found for the CM monocultures. Moreover, we observed that uniaxial cyclic strain could not induce alignment of CMs via solely paracrine signaling of the cFBs or when the cFBs lacked a strain avoidance response. Given this evidence, we hypothesize that uniaxial cyclic strain promotes strain avoidance in cFBs, which, in turn, act as guidance cues for hPSC-CMs (Fig. 6).

Analysis of CMs and cFBs in monoculture and in co-cultures, seeded in a 70/30 or 30/70 (CM/cFB) ratio, on ECM patterns under static conditions revealed that structural cues can effectively guide cellular orientation, resulting in anisotropic cell organization on parallel ECM patterns. This is in line with studies investigating the alignment of cFBs and CMs on patterned FN^11,22^ and other patterned protein substrates (e.g., gelatin,^65^ laminin,^66^ and collagen^66^). It will be interesting for future research to confirm whether this cellular response is evoked primarily by the contact guidance cues in a protein independent manner. Since myocardial tissue mainly consists of parallel collagen I fibers, ECM organization could be a key determinant of cFB and CM organization *in vivo.*^67^

Strikingly, distinct responses were observed for cFBs and CMs when ECM patterns and uniaxial cyclic strain were presented simultaneously. On the anisotropic ECM patterns, both the CMs and cFBs showed only a slight disruption of cellular alignment without a complete strain avoidance response within the 48 h of cyclic strain, supporting the dominance of cellular contact guidance over strain avoidance. It is well-documented that both pattern dimensions and strain magnitude are determinants of cell alignment behavior when cyclic strain and anisotropic protein patterns are presented to cells in competition. This observation is hypothesized to come from the dephosphorylation of focal adhesion intracellular signal before reaching the center of the protein patterns, hampering stress fiber polymerization and leading to a discontinuous activation level of stress fibers perpendicular to the patterns, which results in the inability of cells to form stress fibers in that direction.^68^ In contrast, focal adhesion intracellular signal transmission is not hampered along the direction of the pattern, allowing cells to form long stress fibers parallel to the lines. In line with this, function blocking of adhesion molecules such as integrins and focal adhesion proteins hampered the cellular guidance response by protein patterns.

While cFB monocultures on homogeneous and crosshatch ECM patterns showed a clear and expected strain avoidance response due to the cyclic straining, monocultures of CMs did not. The lack of strain avoidance for CMs presented in this study is in contrast with Qi *et al.*^29^ and Kreutzer *et al.*,^31^ who observed stain avoidance for hPSC-CMs. These inconsistencies might be attributed to the timing of the onset of cyclic strain administration^69^ and to the small focal adhesions (FAs) found in monocultures of CMs or lower expression levels of fibronectin binding integrins,^22^ which could hamper proper transduction of mechanical forces from the microenvironment to the CMs. Moreover, in the present study, we used lower strain rates and straining time than those used in earlier reports, which might affect the strain avoidance response. Also, differences in CM maturity might contribute to the lack of strain-induced alignment since differentiation state is found to influence strain avoidance behavior in cardiomyocyte progenitor cells.^25^ The strain avoidance behavior in our 2D setup might appear to be contradictory to the alignment response in 3D, where cells align in the direction of the applied strain. However, a combination of computational modeling and experimental modeling supported the hypothesis that cell alignment in 3D is primarily dependent on the presence or absence of restraining boundary conditions, instead of the application of strain. Only when higher order strain rates were applied, which reduce stress fiber assembly or promote stress fiber disassembly, strain avoidance in 3D can be found.^70^ Despite the lack of response by CMs, the clear induction of anisotropic organization on the crosshatch patterns for cFBs suggests that uniaxial cyclic strain can be exploited to restore the anisotropic architecture of the myocardium if cFBs dominate the injured area of the myocardium.

Whereas, to our knowledge, no literature exists for human tissues or models on how the amount of cFBs modulates strain avoidance of CMs, a recent study by Tran *et al.* demonstrated a cell density dependent response to cyclic strain for co-cultures of varying ratios of neonatal rat CMs and cFBs.^27^ In contrast to hPSC-derived CMs, monocultures of neonatal rat CMs oriented along the direction of cyclic strain and increasing the amount of cFBs in the co-culture resulted in orientation away from the direction of the applied cyclic strain. However, a higher number of cFBs was needed to induce strain avoidance in neonatal rat CMs as opposed to in co-cultures of human cFBs and CMs derived from pluripotent stem cells, possibly due to differences in maturity and contractile state between the two types of CMs.^27^ Moreover, several studies have investigated the contribution of the amount of cFBs on CM alignment and maturity. For CM maturity, an optimum in contractility was found in co-cultures containing 10%–30% cFBs, which could not be achieved when dermal fibroblasts were used instead of cFBs.^71^ Also, for neonatal rat CMs in cardiac microtissues, a cFB density of 55% or more weakened the CM function, demonstrated by a significant decrease in contractile force.^72^ Interestingly, Rupert *et al.* showed that 15% of cFBs in co-culture with hPSC-CMs in engineered cardiac tissues resulted in less CMs alignment as opposed to 5% of cFBs,^73^ seemingly in contrast to the observations in the present study. However, the experiments performed by Rupert *et al.* were conducted in engineered cardiac tissues where cFBs adopt a more quiescent phenotype as opposed to the contractile phenotype on stiff 2D substrates,^73^ suggesting that the phenotype of cFB may play a role in aligning CMs. These findings demonstrate that a low percentage of cFBs improves CM maturation and tissue contractility. Yet, the diseased myocardial microenvironment consists of mainly contractile cFBs, suggesting that the mechanoresponsiveness of cFBs can be critical in determining CM and overall tissue organization.

Our findings indicate that CMs and cFBs, the main cellular components of the myocardium, differentially respond to structural and mechanical cues that typify the myocardial tissue. Importantly, an interplay between the two cell types is necessary for the overall strain-induced response that leads to anisotropic cell organization as is expected in healthy, contractile myocardium. Therefore, our results propose an intriguing emergence of guidance cues for CMs that are mediated by the mechanoresponsiveness of cFBs; whereas cell orientation can be dynamically induced by topographies and structural guidance cues in *in vitro* culture using light-responsive synthetic materials,^74–76^ we present a similar emergence arising from cues that are inherent in the *in vivo* CM microenvironment—mechanical strain and cFBs. Further investigation should address if the removal of cyclic strain can cease the presentation of the cFB-mediated guidance cues, which would suggest dynamic control over the presentation of these guidance cues. Moreover, future research should investigate if the CMs with increased sarcomere length, organized by cyclic strain, demonstrate stronger contractility, as is expected for aligned CMs as opposed to randomly organized CMs.^11^

Collectively, our data propose the importance of the strain avoidance response of cFBs, a cell type often overlooked in cardiac regenerative strategies, in determining myocardial architecture and function. Moreover, we demonstrate that immature therapeutically promising hPSC-CMs do not respond to the cyclic deformations of the cardiac beating and will align with randomly oriented cFBs. This suggests a prominent role for cFBs in (re)organizing CMs and indicates that more studies should be targeted at aligning cFBs, as far as cardiac organization and repair are concerned. In addition, this knowledge can be relevant when designing cardiac patches that constrain the cardiac wall (e.g., uniaxial constrained tissue allows cFBs to align in the direction of the constraints, which subsequently can align the CMs). Future studies should indicate how the mechanoresponsiveness of cFBs can be exploited in 3D and in *in vivo* heart models as a strategy to regain cardiac anisotropy upon injury. These results not only shed new light on the role of cFBs in structural remodeling upon myocardial damage, but also point at a possible role of cFBs in regaining myocardial anisotropy in the damaged heart. To apply the latter option, computational models should be employed that explore how mechanical strains present in the infarcted myocardial tissue can be used and manipulated to organize the CMs and cFBs along the direction of contraction.^77,78^ Moreover, by exploiting the strain avoidance response of cFBs, the formation of anisotropic structure in engineered tissue constructs can be promoted, aiding the design of strategies for structural organization of the myocardium.

## METHODS

### Fabrication of micro-patterned deformable substrates

Fibronectin (FN) adhesive patterns were transferred onto deformable PDMS membranes using previously established protocols for microcontact printing.^36,79^ In short, a parallel line pattern (10 *μ*m width, 10 *μ*m gap) and a crosshatch pattern (perpendicular crossing with 5 *μ*m wide lines with 10 *μ*m spacing) were generated on a silicon master wafer by deep reactive-ion etching from a chromium photomask.^80^ Microstamps were obtained by molding the silicon master with polydimethylsiloxane (PDMS, Sylgard 184; Dow Corning, Michigan, United States) and curing agent (10:1) and curing the constructs for 20 min at 120 °C. Cured PDMS microstamps with the desired features were then peeled off from the master and cleaned and dried with 70% ethanol and compressed air, respectively. In order to print the protein patterns, the microstamps were inked for 1 h at room temperature with 25 *μ*g/ml rhodamine FN solution (Cytoskeleton Inc., Colorado, USA). Deformable silicone Flexwell membranes (Flexcell Inc., Burlington, USA), supported by an underlying printing mold, served as substrates for the microcontact printing. These substrates were first oxidized in an UV/ozone cleaner (PDS UV-ozone cleaner; Novascan, Iowa, USA) for 8 min, after which the FN-coated stamps (first dried under compressed air) were gently deposited on the substrates and incubated for 15 min at room temperature. Uncoated regions were blocked by immersing the substrates for 5 min in 1% Pluronic F-127 (Sigma-Aldrich, Missouri, USA) in phosphate-buffered saline (PBS). Finally, the membranes were washed three times with phosphate-buffered saline (PBS, Sigma-Aldrich) and stored in PBS at 4 °C until further use. A microstamp without any features was used to print homogeneous FN as a control substrate.

### Human induced pluripotent stem cell derived cardiomyocyte culture

Human induced pluripotent stem cell derived cardiomyocytes (hiPSC-CMs) were differentiated according to the protocol described before^81^ and kindly provided by Professor E. van Rooij (Hubrechts Institute, Utrecht, the Netherlands). hiPSC-CMs were maintained in RPMI 1640 with 4-(2-hydroxyethyl)-1-piperazineethanesulfonic acid (HEPES) and Glutamax (Life Tech, Bleiswijk, the Netherlands) with 2% B27 with insulin (Life Tech) and 1% penicillin/streptomycin (P/S, Sigma-Aldrich). Six days before mechanical stimulation, hiPSC-CMs were plated on the micropatterned deformable substrates in order to allow cryopreservation recovery and spontaneous beating retrieval. Cells were seeded at 50 000 cells/cm^2^.

### Human pluripotent stem cell derived cardiomyocyte culture (DRRAGN)

The DRRAGN hPSC (derived from human embryonic stem cell line HES3) line 3F4, modified with a double reporter of GFP-NKX2.5 and mRubyIIACTN2, was kindly provided by Professor R. Passier (University of Twente, the Netherlands). The hPSCs were cultured on vitronectin-coated culture plates and maintained by refreshing Essential 8 (E8) medium (Thermo Fisher, Waltham, Massachusetts, USA) daily and passaging the cells at around 150k cells/cm^2^ each Monday and Thursday. The hPSCs were differentiated into cardiomyocytes (hPSC-CM), according to the protocol described before.^82^ On day 16 of differentiation, the cells were dissociated and cryopreserved in 50% knock-out serum, 40% bovine serum albumin polyvinyl alcohol essential lipids (BPEL) medium, 10% dimethylsulfoxide (DMSO), and rock inhibitor (Y27632, 1:200). The hPSC-CMs were thawed five days before the start of each experiment.

### Epicardial-derived cardiac fibroblast culture

Human epicardial-derived cardiac fibroblasts (cFBs) were kindly provided by Professor M. J. Th. H. Goumans (Leiden University Medical Center, the Netherlands). cFBs were isolated from human fetal cardiac tissue collected with informed consent and anonymously from elective abortion material of fetuses with a gestational age between 10 and 20 weeks. This research was carried out according to the official guidelines of the Leiden University Medical Center and approved by the local Medical Ethics Committee (No. P08.087). cFBs were cultured in high-glucose Dulbecco's modified Eagle's medium (Invitrogen, Breda, the Netherlands) supplemented with 10% fetal bovine serum (Greiner Bio-one) and 1% P/S (fibroblast culture medium). The cFBs were cultured in flasks coated with 0.1% gelatin from porcine skin in PBS (Sigma-Aldrich) and passaged at 80% confluency. The passage number of the cFBs during mechanical stimulation was between 4 and 8.

### Cardiac co-cultures

In order to analyze the contribution of cFBs to the alignment response of CMs, co-cultures were created with varying cell seeding densities. Either hiPSC-CMs or DRRAGN CMs were dissociated with 10× TrypLE (Gibco, Eindhoven, the Netherlands) and seeded in maturation medium (TDI) 48 h prior to mechanical stimulation (day-2). After 24 h (day-1), the cFBs were dissociated using 0.05% Trypsin/Ethylenediaminetetraacetic acid (EDTA) (Gibco, Eindhoven, the Netherlands) and added to hPSC-CMs in co-culture medium containing 25% fibroblast culture medium and 75% TDI medium. 150 *μ*l of cell suspension was seeded only on top of the coated region of the flexible PDMS substrates and incubated at 37 °C overnight to allow local cell adherence before the extra co-culture medium was added. The co-cultures were maintained in hPSC-CM medium and incubated at 37 °C and 5% CO_2_ until analysis. Monocultures of hPSC-CMs and cFBs were seeded onto the same substrates and mechanically stimulated as control.

### Cyclic straining of mono and co-cultures

The FX-5000^TM^ Tension System (Flexcell Inc., Burlington, USA) was used to provide controlled uniaxial cyclic strain to the cardiac cells cultured on the flexible membranes by means of a regulated vacuum pressure that deforms the membrane. The cardiac cultures were seeded one or two days prior to straining to allow for cell adhesion and cellular organization through the printed fibronectin patterns (d_1_) and were strained for 48 h thereafter. Strains of 10% (0.5 Hz, sine wave) were applied to the cardiac cultures, increasing stepwise from day 0 to condition the cells, and reaching 10% strain after 8 h. On day 0 (before the onset of strain) and after 48 h (day 2), the samples were collected. Unstrained cardiac mono and co-cultures were used as control. The cyclic strain applied to the membranes in 2D was validated using image analysis. First, graphite powder was sprayed on top of membranes that were excluded from cell culture, several stretching cycles were recorded by a camera, and the applied cyclic strain was measured by the displacement of the graphite pattern with ImageJ.

### Cardiac fibroblast conditioned medium

cFB conditioned medium was obtained from cFBs after 48 h of mechanical uniaxial cyclic strain to allow secreting of paracrine factors. 50 000 cFBs were plated on homogenous substrates 24 h before mechanical stimulation. 3 ml cFB culture medium (as described in 2.3) was added to every well, and the cFBs were strained (as described in 2.6) for 48 h. After mechanical stimulation, the conditioned medium was retrieved from the cFBs and filtered using a 0.2 *μ*m syringe filter to remove cells and other large particles. The medium was frozen immediately and thawed just before it was mixed 1:1 with fresh TDI medium and added to monocultures of 100 000 hPSC-CMs, which were mechanically loaded or statically cultured as control.

### Viability assay

To verify the coexistence of hPSC-CMs and cFBs in the co-cultures, cell viability was assessed using calcein AM and propidium iodide (Invitrogen) one day after seeding (day 0) and 48 h after that (day 2). In short, the co-cultures were carefully washed with PBS and incubated with 1 *μ*g/ml calcein AM and 750 nM propidium iodide for 20 min at 37 °C protected from light. After incubation, the cultures were washed with PBS directly imaged with an inverted fluorescence microscope (Zeiss Axiovert 200m). Images were taken at three random representative spots per condition. Three individual experiments were performed with 2–3 replicates (n = 2–3), and co-cultures treated with ethanol were used as negative control.

### Quantification of cell orientation

The orientation response of the cardiac cells was determined from triplicates of three independent experiments (n = 3). Cells were incubated with 1 *μ*g/ml of live cell tracker calcein AM (Sigma-Aldrich) for 20 min and visualized after medium renewal with an inverted microscope (Zeiss Axiovert 200m equipped with an AxioCam HR camera; Zeiss, Sliedrecht, the Netherlands). Only the central parts of the wells, where the strain field was homogeneous as determined using our in-house calibrations, were considered (1 cm^2^, 50 000 cells). In total, the orientations of ∼250 cells were analyzed per condition. Cell orientation was quantified with ImageJ, using the Fiji plug-in “directionality,” based on Fourier spectrum analysis (Fig. S6). In short, fluorescence images were converted to eight-bit images, and directionality analysis was performed based on the fast Fourier transform (FFT) of each image using a Gaussian fit to the FFT signal. After choosing a bin size (2°), the algorithm provides the oriented element fraction per bin in a specified region of interest (ROI, 320 × 320 *μ*m^2^), and these values were exported to Graphpad Prism software (version 5.04) to visualize graphically.

### Immunofluorescence staining

The cardiac cells cultured on the Bioflex plates were washed with PBS trice before and after fixation and fixed in 3.7% formaldehyde (Merck, Darmstadt, Germany) for 15 min. The flexible membranes were cut out of the Bioflex plates and subsequently cut in quarters to allow multiple analysis per well. The cells were permeabilized with 0.5% Triton-X-100 (Merck) in PBS for 10 min and blocked for nonspecific antibody binding with 10% horse serum (Sigma-Aldrich) in PBS for 40 min. The cells were incubated overnight with the primary antibodies at 4 °C. The cells were washed six times with PBS for 5 min before incubating with the secondary antibodies in PBS and phalloidin for 1.5 h. All used primary and secondary antibodies and dyes are listed in Table S1. The cells were washed two times with PBS before incubating with DAPI in PBS for 5 min to visualize the cell nuclei. Finally, cells were washed four times with PBS for 5 min, and thereafter, the membranes were mounted to glass slides with Mowiol (Sigma-Aldrich) and stored protected from light at 4 °C. All immunofluorescent samples were analyzed with a confocal fluorescence microscope (Leica SP5X) using the 10× and 20× objectives.

### Quantification of F-actin orientation and sarcomere orientation and length

The orientation response of the F-actin stress fibers and sarcomere structures was determined from triplicate of three independent experiments (n = 3). Immunofluorescent samples were analyzed with a confocal fluorescence microscope (Leica SP8X). In total, the F-actin and sarcomere orientations of ∼250 cells were analyzed per condition. The F-actin and sarcomere orientation were quantified with MATLAB using the in-house developed Fiber Orientation Analysis (FOA) Tool, where F-actin and sarcomere structures are identified using Frangi vesselness. Sarcomere length was quantified with ImageJ as the distance between adjacent α-actinin bands in confocal images. Spacing of individual α-actinin bands (n = 500 per condition) was collected from images from 10 to 15 cells in three different batches.

### Statistical analysis

All results are expressed as mean ± standard error of the mean (SEM) (n = 3 with three technical replicates per group), unless indicated otherwise. Statistical analysis was performed using Wilcoxon matched-pairs signed rank test for non-parametric two group comparison. For multiple group comparison, one-way analysis of variance (ANOVA) was used with Bonferroni's post-hoc test. Significance was assumed when p < 0.05. Graphics and statistical analyses were performed with Graphpad Prism software (version 5.04).

## SUPPLEMENTARY MATERIAL

See the supplementary material for additional figures concerning method validation and the effect of paracrine signaling and N-cadherin mediated interactions on the strain responsiveness of CMs.

## Data Availability

The data that support the findings of this study are available from the corresponding author upon reasonable request.
